# A Foreign Body-Induced Duodenal Perforation Complicated by Retroperitoneal Abscess and Necrotizing Pancreatitis: A Report of a Rare Case

**DOI:** 10.7759/cureus.96364

**Published:** 2025-11-08

**Authors:** Anjani Mahesh Kumar Cherukuri, Shikha Magar, Katarina Valentic, Melanie Quiñones-Candelaria, Rasheed Fontenelle, Hyeonji Lee, Minjune Song, Mikolaj Dulaj, Adam Durrani, Priscilla Evellin de Azevedo Torres, Raul Mederos

**Affiliations:** 1 Surgery, Guntur Medical College, Guntur, IND; 2 Surgery, Kempegowda Institute of Medical Sciences, Bengaluru, IND; 3 Surgery, American University of Antigua, College of Medicine, Osbourn, ATG; 4 Surgery, University of the West Indies, Mona, JAM; 5 Surgery, Kyung Hee University, School of Medicine, Seoul, KOR; 6 Surgery, Kyungpook National University, School of Medicine, Daegu, KOR; 7 Surgery, Medical University of Lodz, Lodz, POL; 8 Medicine, Royal College of Surgeons in Ireland, Busaiteen, BHR; 9 Surgery, University of Ribeirão Preto, São Paulo, BRA; 10 Surgery, Hialeah Hospital, Hialeah, USA

**Keywords:** acute necrotising pancreatitis, duodenal perforation, foreign body perforation, gastrointestinal perforation, retroperitoneal abscess

## Abstract

Duodenal perforations are rare but critical conditions that often present with significant morbidity and mortality. These injuries may result from peptic ulcer disease, trauma, or foreign bodies, with posterior perforations posing particular challenges due to their retroperitoneal location. Such perforations may lead to complications, including retroperitoneal abscesses, necrotizing pancreatitis, and fistula. The management of these cases often requires a multidisciplinary approach. This case report describes a highly unusual instance of a complicated posterior duodenal perforation in a 76-year-old male, caused by a foreign body, which was later identified as an aggregate of multiple fragments of vegetable matter, necessitating surgical intervention. This case highlights the importance of recognizing atypical causes of perforation and underscores the complexity of managing such rare occurrences. By presenting this case, we aim to contribute to the growing body of literature on the topic and discuss the diagnostic, therapeutic, and clinical implications of managing such cases.

## Introduction

The ingestion of foreign bodies is a frequent occurrence in pediatric populations, individuals with alcohol dependency, and patients with psychiatric disorders. In adults, particularly in edentulous elderly individuals, such incidents are typically the result of involuntary or accidental ingestion of food-related foreign objects. It is estimated that approximately 80% of ingested foreign bodies pass through the gastrointestinal tract and are excreted without leading to significant complications [1]. However, 10% to 20% of cases necessitate endoscopic removal, while fewer than 1% require surgical intervention either for foreign body extraction or to address associated complications [1,2].

Foreign body impaction, obstruction, or perforation most commonly occurs at areas of anatomical narrowing or sharp angulations within the gastrointestinal tract, with the upper esophagus being the most frequent site of lodgment. Although rare, foreign bodies can also become lodged in the duodenum, potentially causing serious complications such as perforation, obstruction, abscess, peritonitis, pancreatitis, and fistula formation [3,4]. The clinical presentation is often nonspecific, making diagnosis challenging.

A serious complication is retroperitoneal abscess formation, requiring antibiotics and drainage [5]. In severe cases, necrotizing pancreatitis may develop, necessitating intensive management and potentially surgery [4]. Delays in treatment can significantly increase morbidity, and perforations in this region can be life-threatening, with reported mortality rates ranging from 8% to 25% [6].

Given the potential for severe complications, effective management is imperative in cases of duodenal perforation. Contained perforations may be managed conservatively, whereas patients with non-contained perforations and progressive deterioration of their condition necessitate surgical intervention. We present a case in which initial conservative management was considered, but due to the patient's worsening condition, surgical intervention was ultimately necessary.

## Case presentation

A 76-year-old male with a past medical history of hypertension controlled with medications, chronic smoking, and a surgical history of left inguinal hernia repair presented with an acute onset of epigastric pain and three episodes of non-bilious, non-bloody emesis associated with nausea and chills. On arrival, vital signs were remarkable for blood pressure of 158/87 mmHg, oxygen saturation of 93% on room air, and a BMI of 62.3 kg/m^2^. Physical exam revealed mild rhonchi at the lung bases without respiratory distress, and diffuse tenderness to palpation of the abdomen with no rebound and no guarding. Laboratory investigations, as shown in Table 1, included leukocytosis (14.3 x 10*9/L) and a markedly elevated lipase (27,000 U/L), suggestive of pancreatic involvement.

**Table 1:** Comprehensive laboratory findings on admission, including inflammatory markers and pancreatic enzymes. POD: postoperative day; AST: aspartate aminotransferase; ALT: alanine aminotransferase.

Investigation	On admission	POD 0	POD 1	Reference	Units
RBC	5.42	5.44	4.89	4.63-6.08	x10^6^ mcL
WBC	14.3	-	14.8	5.2-10.0	10*3/uL
Lipase	27771	57959	6787	23-300	U/L
Amylase	-	>2400.0	1182.0	30.0-110.0	U/L
Total bilirubin	0.70	3.30	6.40	0.20-1.30	mcg/dL
AST	47	258	538	17-59	U/L
ALT	32	123	330	21-72	U/L
pH	7.45	7.27	7.26	7.35-7.45	-
Lactate	2.1	7.0	3.8	0.7-2.0	mmol/L
Sodium	139	142	144	137-145	mmol/L
Potassium	3.9	4.6	3.4	3.4-5.0	mmol/L
Chlorine	105.0	109.0	113.0	98.0-107.0	mmol/L

**Figure 1:**
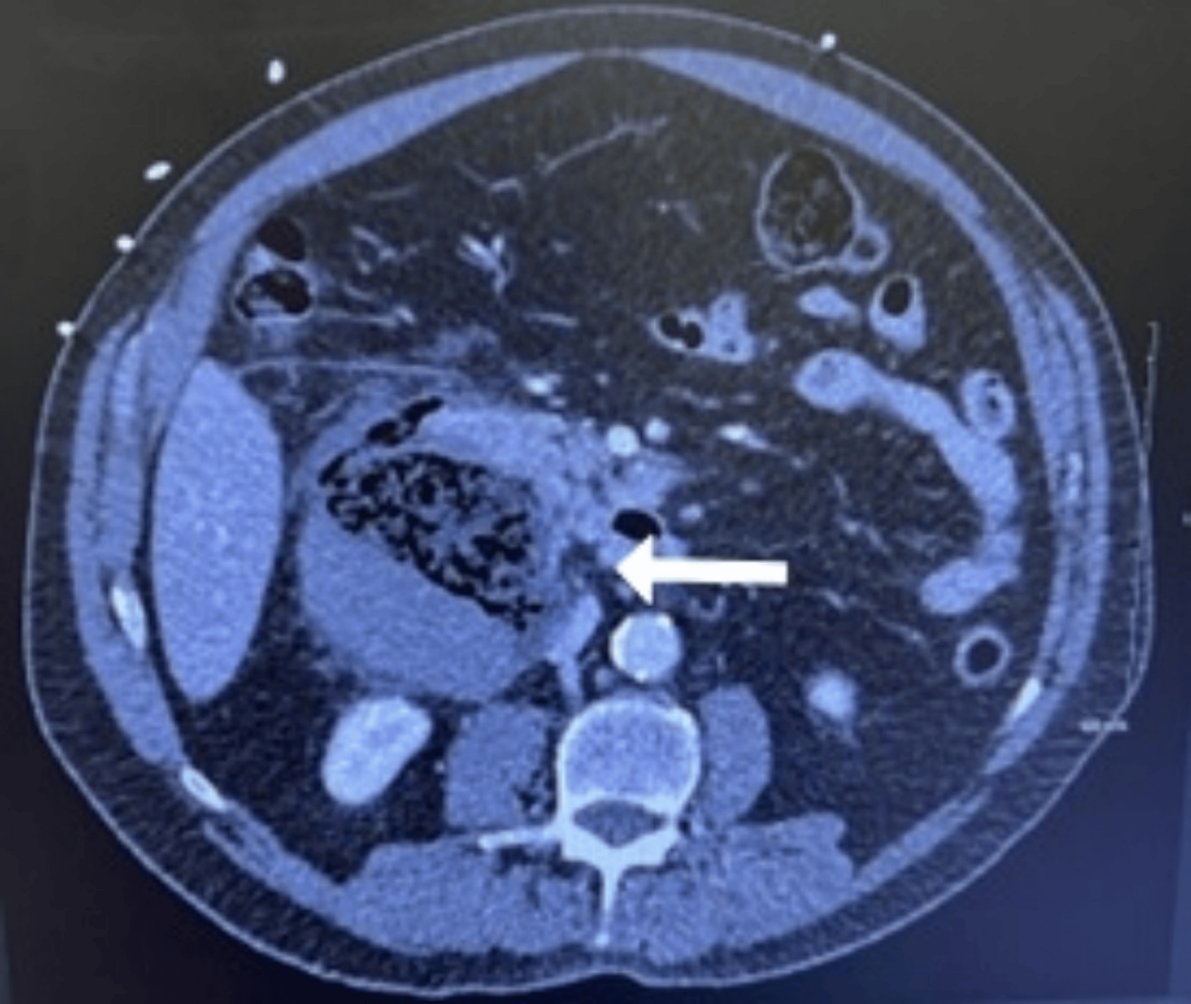
Abdominal CT in axial view with contrast showing a large right retroperitoneal complex fluid collection.

**Figure 2:**
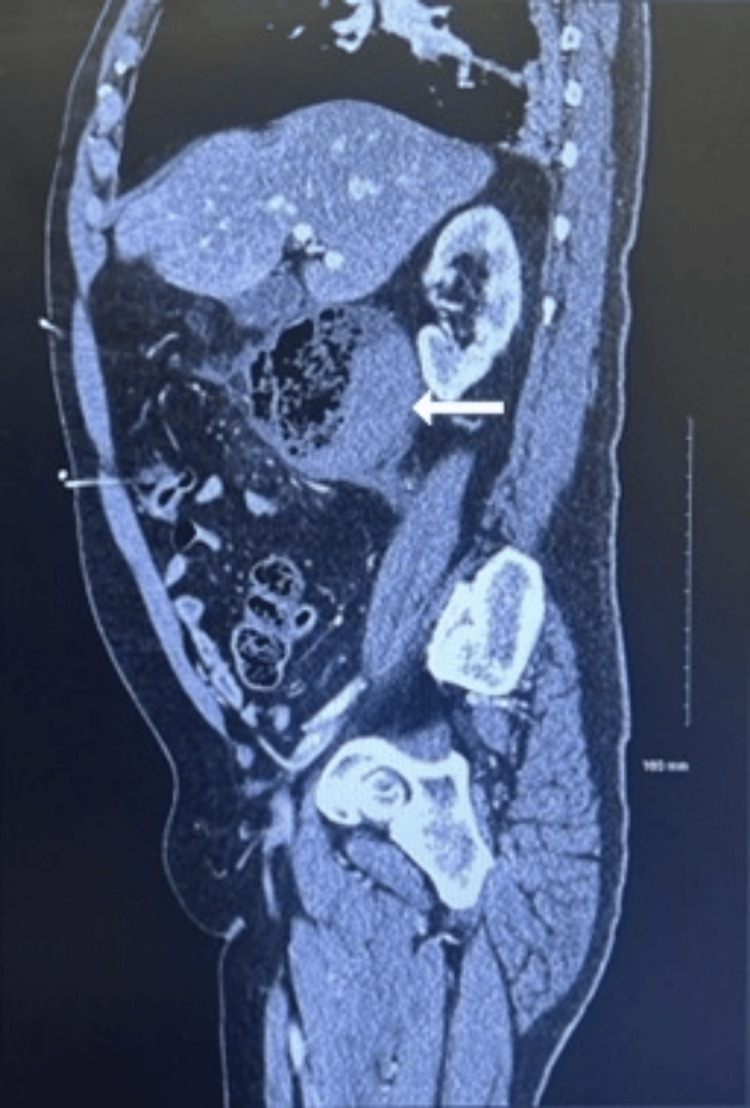
Abdominal CT in sagittal view.

**Figure 3:**
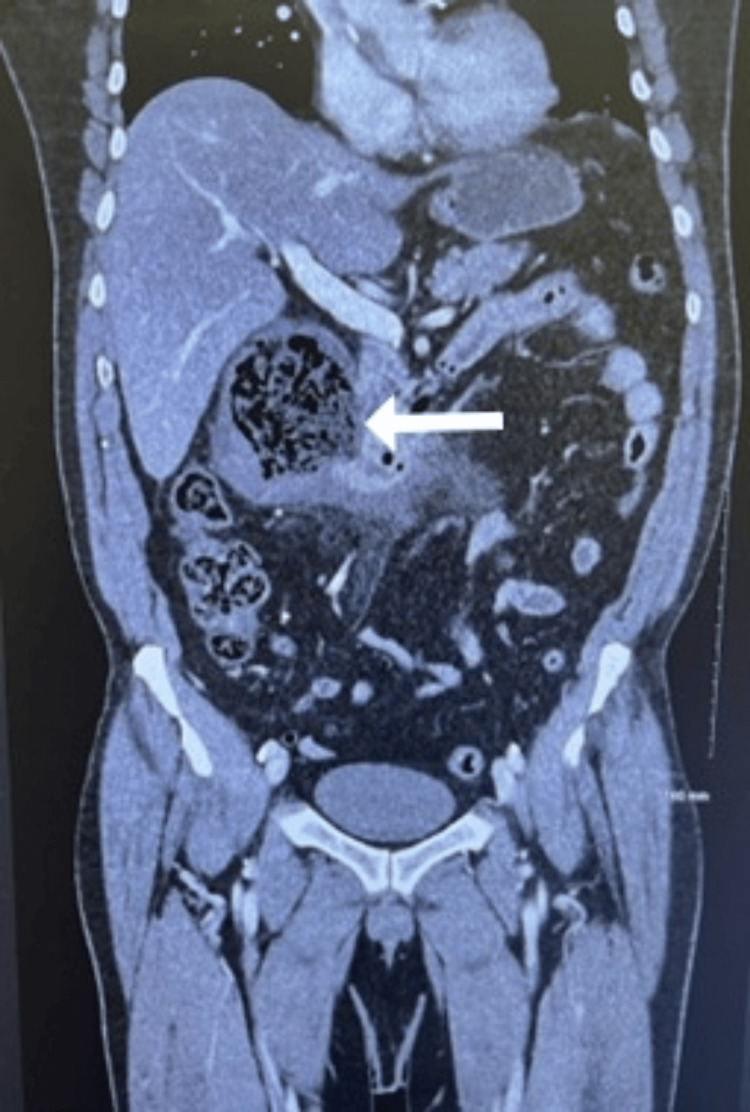
Abdominal CT in coronal view.

The CT scan of the abdomen/pelvis with contrast showed a large right retroperitoneal complex fluid collection with wall thickening and fat stranding just posterior to the second portion of the duodenum, measuring 7.7 x 8.1 cm, as seen in Figures 1-3. The imaging suggested a contained duodenal perforation, with no evidence of free air or free fluid. The patient was admitted on the same day for aggressive IV fluid resuscitation, antibiotics, and stabilization and optimization with a nasogastric tube and nil per oral (NPO) order. Despite trial of conservative management, the patient rapidly developed progressive respiratory failure, severe acute mental status changes, and other signs of multiorgan system failure, prompting emergency surgical intervention within 24 hours of admission. The patient underwent an exploratory laparotomy, which revealed a large retroperitoneal abscess posterior to the second portion of the duodenum. The abscess contained necrotic debris, hard food particles, and what appeared to be vegetable material resembling sticks extruding from a large defect in the posterior duodenal wall. The defect involved a disruption of over 75% of the circumference of the posterior duodenal wall. The retroperitoneal abscess extended into the head of the pancreas, necessitating extensive evacuation of necrotic material from both the abscess cavity and the pancreatic region.

Completion of the surgery began with the primary repair of the posterior duodenal perforation. Next, pyloric exclusion was performed, and a gastrojejunostomy was created. After irrigation, the surgeon proceeded with closure, and the patient was taken to the ICU for ongoing resuscitation and close monitoring.

**Figure 4:**
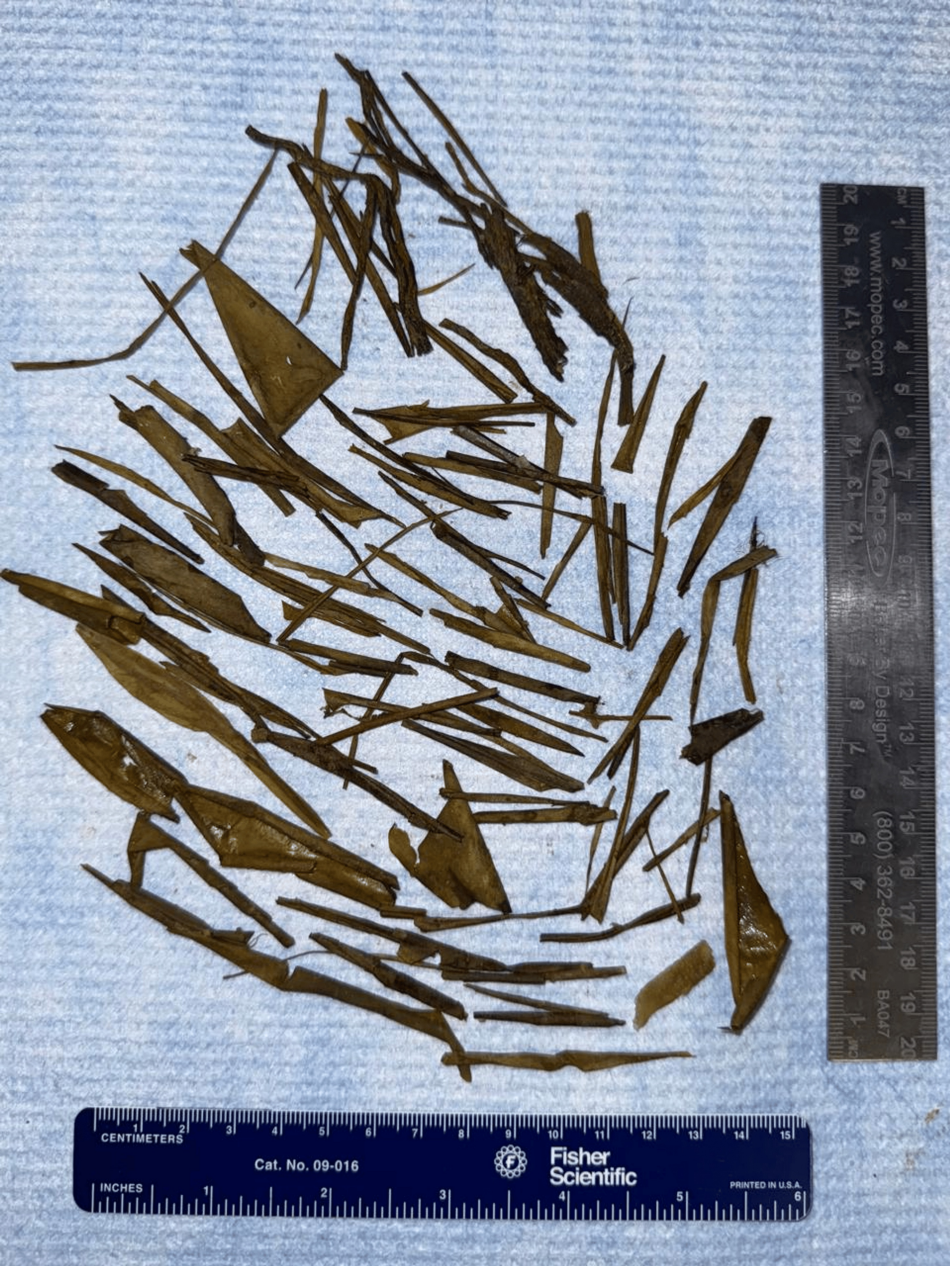
Foreign body.

Pathology reported the gross description of the specimen received as an aggregate of multiple fragments of vegetable matter measuring 9 x 7 x 2.7 cm with no tissue identified, as seen in Figure 4. No slides were prepared, and microscopic examination was not performed.

Culture reports on the retroperitoneal abscess indicated heavy growth of *Escherichia coli*. The retroperitoneal abscess exhibited light growth of *Escherichia coli*. Samples taken from the body fluid were remarkable for the growth of two organisms, *Citrobacter koseri* and *Escherichia coli*.

Postoperative course and management

The patient was transferred to the ICU following surgery for close monitoring and ongoing resuscitation. At the time of admission to the ICU, the patient remained intubated and in critical condition. He exhibited persistent hemodynamic instability and signs of severe sepsis, likely secondary to retroperitoneal contamination and systemic inflammatory response from the duodenal perforation. Broad-spectrum antibiotics were continued, and supportive measures were escalated, including vasopressor support and renal monitoring.

Despite aggressive management, the patient failed to regain consciousness in the immediate postoperative period. Neurological status showed no improvement, and he remained comatose and unresponsive to external stimuli. His clinical course was further complicated by progressive multiorgan dysfunction, with steadily declining urine output and worsening renal parameters, consistent with acute kidney injury.

Given the severity of his septic state and the poor neurological prognosis, the family was counseled extensively. After thoughtful discussions regarding the patient's lack of recovery and deteriorating condition, the family made the difficult decision to withdraw life-sustaining interventions. The patient was transitioned to comfort-focused care and passed away shortly thereafter.

## Discussion

Foreign body ingestion is a common emergency worldwide, with most objects passing through the gastrointestinal tract within a week without complications [7]. The size and structure of the foreign body can influence the severity of complications, which may range from mild abdominal pain to obstruction, perforation, hemorrhage, and fistula formation [3,4]. High-risk groups include geriatric patients with dentures, those with previous gastric bypass surgery, individuals with abdominal adhesions, and patients with sensory deficits following cerebrovascular accidents [8].

Bowel perforation is rare (<1%) and is more likely with longer or sharper foreign bodies, typically occurring at areas of narrowing or anatomical angulations, such as the ileocecal junction, sigmoid colon, and duodenojejunal flexure [7,8]. Duodenal perforations may result from duodenal pathology, iatrogenic causes, trauma, foreign bodies, or spontaneously [6]. In a study by Ma et al. evaluating 239 duodenal foreign bodies, 15.9% led to perforation, a rate significantly higher than the overall GI perforation rate of 1.4% [1].

While perforations caused by dietary foreign bodies, such as fish bones, chicken bones, and toothpicks, are relatively common, those resulting from vegetable matter are exceptionally rare [1,8,9] Notably, there have been only a few documented instances of small bowel perforation attributed to vegetable content (tomato peel) and the occurrence of duodenal perforation due to vegetable matter appears to be extremely uncommon, making this case particularly notable for the literature [10].

Symptoms can range from mild epigastric discomfort to severe abdominal pain, nausea, vomiting, and signs of peritonitis or sepsis in more severe cases [4]. Due to the retroperitoneal location of the duodenum, perforation in these areas may lead to retroperitoneal contamination rather than free intraperitoneal air, rendering standard radiographic findings unreliable [5]. As a result, CT with contrast has become the preferred imaging modality for detecting duodenal perforations and complications.

Duodenal perforations can lead to chemical peritonitis due to the leakage of intraluminal contents, potentially initiating a systemic inflammatory response syndrome (SIRS). This may subsequently progress to bacterial peritonitis and sepsis. Other possible complications include retroperitoneal abscesses, migration of the foreign body, and perforation into adjacent organs [9].

Retroperitoneal abscess formation is a significant complication, often polymicrobial, and requires broad-spectrum antibiotics along with drainage [5]. In severe cases, the inflammatory process can extend to the pancreas due to the proximity of the duodenum and pancreatic head, resulting in necrotizing pancreatitis [4]. This condition typically demands aggressive management, including fluid resuscitation, intensive care, and potentially surgical or endoscopic necrosectomy. Both of these complications were observed in our case, highlighting the severity and complexity of the condition.

Treatment generally includes surgical intervention, such as primary closure with or without the application of an omental patch, or the use of pedicled omental flaps (Cellan-Jones) or free omental plugs (Graham patch) [6]. Sutureless techniques with gelatin sponge and fibrin glue have also been employed. There are no significant differences in postoperative morbidity and mortality between these methods. Repair can be done via open surgery or laparoscopy, with laparoscopic approaches offering reduced postoperative complications and shorter hospital stays. The management of duodenal perforations remains a subject of ongoing debate, largely influenced by observational data and case reports [6]. Key debates include the role of non-operative management, surgical approach, and the use of gastric diversion procedures.

This case of duodenal perforation due to vegetable content contributes valuable insight to the limited literature on such rare occurrences, emphasizing the need for further exploration and discussion of management strategies in similar cases.

## Conclusions

In conclusion, duodenal perforation due to foreign body ingestion, particularly from vegetable content, remains a rare and underreported phenomenon. This case highlights the importance of recognizing atypical causes of perforation and underscores the complexity of managing such rare occurrences. While current treatment strategies predominantly involve surgical intervention, the optimal management approach remains a subject of ongoing debate, with a need for further research to refine treatment protocols and improve patient outcomes. This case contributes valuable data to the limited literature on foreign body-induced duodenal perforations. Future studies should focus on refining diagnostic algorithms, optimizing treatment approaches, and improving strategies for preventing complications.
